# “It’s Just a Matter of Doing It”: Women Veterans’ Experiences with Changing Behavior for Cardiovascular Risk Reduction

**DOI:** 10.1007/s11606-026-10306-9

**Published:** 2026-04-20

**Authors:** Kimberly Clair, Erica H. Fletcher, Anneka Oishi, C. Amanda Schweizer, LaShawnta Jackson, Catherine Chanfreau, Erin P. Finley, Alison Hamilton, Tannaz Moin, Melissa M. Farmer, Bevanne Bean-Mayberry

**Affiliations:** 1 Center for the Study of Healthcare Innovation, Implementation & Policy (CSHIIP), VA Greater Los Angeles Healthcare System, Los Angeles, CA USA; 2 Departments of General & Hospital Medicine & Psychiatry, University of Texas Health Science Center, San Antonio, TX USA; 3 Department of Psychiatry and Biobehavioral Sciences, David Geffen School of Medicine, University of California Los Angeles, Los Angeles, CA USA

**Keywords:** women’s health, cardiovascular disease, lifestyle change, veteran health

## Abstract

**Background:**

Cardiovascular disease (CVD) is the leading cause of death among women in the U.S., and prevalence rates are even higher among women Veterans. While behavioral interventions, such as improving diet and increasing exercise, are widely recommended to reduce CVD risk, implementing and sustaining such lifestyle changes can be challenging.

**Objective:**

We characterized women Veterans’ experiences making lifestyle changes to reduce their CVD risk, including self-reported barriers and strategies that facilitated success.

**Design:**

A quality improvement initiative to increase women Veterans’ engagement and retention in evidence-based health care for CVD risk reduction (the CV Toolkit) was implemented across five VA primary care sites June 2017–March 2020. We conducted semi-structured interviews with a sub-sample of women Veterans and used directed content analysis to identify themes.

**Participants:**

Women Veterans with at least one primary care visit and exposure to at least one component of the CV Toolkit (*N* = 1609) were invited to complete a mailed survey; among survey completers (*n* = 253), 48 participated in a 30-min interview about their experiences.

**Main Measures:**

We assessed lifestyle change type, barriers, and experiences implementing these changes.

**Key Results:**

Women Veterans reported making changes to diet, exercise, smoking, sleep, mental health, and medication adherence. Barriers included physical health, mental health, transportation, work schedule, care-giving responsibilities, limited access to resources/information, the COVID-19 pandemic, and personal attributes. Strategies that helped women Veterans be successful included the following: taking an incremental approach; setting small, achievable goals; and implementing targeted provider recommendations. Women also reported achieving success through self-education, finding a compelling motivation, and practicing mindfulness techniques.

**Conclusions:**

Women Veterans expressed considerable self-awareness regarding strategies that helped them successfully implement lifestyle changes to reduce CVD risk. Providers can leverage women Veterans’ self-awareness to develop personalized, realistic lifestyle change strategies that promote adherence and long-term improvements to cardiovascular health.

## BACKGROUND

Cardiovascular disease (CVD) is the leading cause of death among women in the U.S.^1,2^ with a higher prevalence rate seen among women Veterans.^3^ Introducing lifestyle changes, such as increasing physical activity, improving diet, improving sleep, and eliminating smoking, is widely recommended by the American Heart Association, the VA National Center for Health Promotion and Disease Prevention, and the Centers for Disease Control and Prevention to reduce the risk of obesity, Type 2 diabetes, and other CVD risk factors.^4–6^ Although 80% of chronic conditions can be prevented by the adoption of healthy lifestyle behaviors,^7^ patient-specific barriers to adopting and sustaining evidence-based lifestyle changes remain.

Women Veterans face multiple barriers to making lifestyle interventions, including competing demands such as balancing work and caregiving responsibilities,^8,9^ transportation and other access limitations to exercise programs and facilities,^10^ and a preference for female-specific programs.^11^ Women Veterans with cardiovascular conditions often have comorbid mental health conditions, such as anxiety, post-traumatic stress disorder (PTSD), and depression,^12,13^ which may create additional barriers to successful lifestyle change implementation.^14,15^ Yet gaps remain in knowledge of women Veterans’ perceptions of how these barriers may affect their uptake and consistent participation in evidence-based lifestyle change interventions.

## OBJECTIVE

We characterized women Veterans’ experiences making lifestyle changes to reduce their CVD risk, including barriers to making changes and strategies that facilitated success. Understanding patient-reported challenges and success strategies can inform the development of tailored interventions that promote lifestyle change adherence and long-term improvements to women Veterans’ cardiovascular health.

## METHODS

### Study Design and Participants

A quality improvement initiative to increase women Veterans’ engagement and retention in evidence-based health care for CVD risk reduction—the CV Toolkit^16^—was implemented across five geographically diverse VA primary care sites from June 2017 to March 2020. The CV Toolkit was comprised of three key components: a patient-facing, one-page CV screener, given by the clerk or nurse to women at check-in for their primary care appointment; a provider CV template that mirrored the patient screener to facilitate CV risk discussion and allowed providers to document directly into the electronic health record (EHR); and a menu of appropriate health promotion/behavioral change programs available at each site, to assist providers with on-the-spot referrals.^16^ Women Veterans with at least one primary care visit and exposure to at least one component of the CV Toolkit intervention (*N* = 1609) were invited to complete a mailed survey. Among those who completed the survey (*n* = 253), which collected demographic data and domains pertaining to CV risk, 196 women Veterans expressed interest in being interviewed and provided complete contact information on the survey. Forty-eight participated in a 30-min interview about their experiences making lifestyle changes. Participants who were interviewed did not differ significantly in age, race, or educational background from survey completers who were not interviewed (*n* = 253).^17^ Informed consent was obtained verbally from participants at the start of each interview.

### Data Collection and Analysis

Our semi-structured interview guide inquired about the type of lifestyle changes women Veterans wanted to make, barriers to making lifestyle changes, and their experiences implementing such changes, as well as other domains not included in this analysis (e.g., knowledge of and attitudes towards CVD risk; recent experiences with CVD risk). Demographic data were not collected for this qualitative analysis. Telephone interviews were conducted by our qualitative team consisting of interviewers with graduate-level training in health services research. Interviews were recorded and professionally transcribed.

Anonymized interview transcripts were uploaded into ATLAS.ti software^18^ and analyzed using directed content analysis.^19^ A codebook was developed by the lead author and an analyst (KC, AO): topical codes were informed by the interview guide (change type, barriers, experiences), while subcodes were developed through constant comparison analysis and iterative discussion of emerging themes.^20,21^ After achieving coding consistency among coders (KC, EHF) and with consensus from all qualitative team members, the final codebook was applied to the full sample of interviews. Coding was divided between two team members (KC, EHF). Reported lifestyle changes were tallied among the 48 interviews to assess frequency and type.

Informed consent was obtained from all research participants. This multi-site study received all necessary ethical approvals prior to the start of the study and was approved by the VA Greater Los Angeles institutional review board.

## KEY RESULTS

### Participant Characteristics

Women Veterans had a mean age of 58, with 41% of participants age 65 or older (Table 1). Nearly one-third of participants were non-White (31%) and married (30%) while more than half (57%) had a bachelor’s degree or higher. Nearly half (48%) were employed and 24% reported having care-giving responsibilities for at least one child or adult. Women Veteran participants reported high rates of depression (61%), high blood pressure (54%), high cholesterol (44%), and obesity/overweight (72%).

**Table 1:** Women Veterans’ Demographic and Health Characteristics (*N* = 46, Missing Data for 2 Participants)

Age (M, SD)	58.0 (12.0)
Age 65 +	19 (41.3%)
Race (non-White)	12 (30.8%)
Marital status (married)	14 (30.4%)
Education (Bachelor’s degree or higher)	26 (56.5%)
Employment status (employed)	22 (47.8%)
Care-giving (provided regular non-paid care for ≥ 1 child/adult)	11 (24.4%)
Health conditions (self-reported)	
Depression	28 (60.9%)
High blood pressure	25 (54.4%)
High cholesterol	20 (43.5%)
Obese/overweight	33 (71.7%)
At least 1 CV risk factor^+^	42 (91.3%)

^+^Of 3 CV risk factors (high blood pressure, high cholesterol, obese/overweight)

### Lifestyle Change Types

Women Veterans reported making a variety of behavior changes, including changes to diet, exercise, smoking, sleep, mental health, and medication adherence. Most women (83%) reported attempting more than one health behavior change. Improving diet (75%) and increasing exercise (75%) were the most reported changes. More than one-third of women (35%) reported using a program to make the behavior change; of those, more than half participated in MOVE!, a VA-sponsored weight management program.^22^

### Barriers

Women Veterans described a wide range of barriers to making lifestyle changes, including physical health conditions, mental health issues, transportation, work schedule, care-giving responsibilities, limited access to resources/information, the COVID-19 pandemic, and personal attributes (e.g., inconsistency, lack of motivation, lack of discipline) (Table 2). Physical health issues included injuries and musculoskeletal conditions, sleep disturbances, pain, gastrointestinal issues, polycystic ovary syndrome (PCOS), blindness, seizures, and conditions related to aging (e.g., osteoporosis, physical frailty). These conditions made it difficult for women Veterans to engage in or increase physical activity or to implement healthy eating guidelines while simultaneously managing food sensitivities or digestive symptoms. Many women Veterans described being physically active in the past but unable to continue with their preferred form of exercise due to deteriorating health, injuries or pain. One Veteran explained, “I’m wanting to increase my exercise, but I do have arthritis and fibromyalgia and stuff like that so I have to ask my primary if it’s okay to increase because I’m older now…I don’t want to hurt myself, and I have osteoporosis.” For some women, being overweight was its own barrier to weight loss, reducing their overall energy and making exercise more difficult.

**Table 2:** Women Veterans’ Self-reported Barriers to Implementing Lifestyle Changes

Barrier	Exemplary quotes
Physical health	“I used to get a great deal of exercise. But now I’m having trouble walking, it’s harder to figure out what kind of exercise to do.” “You put on more weight, and it gets harder to exercise because you’re carrying more weight around, and your knees hurt, and your back hurts.”
Mental health	“I deal with major depression, and so I have other things healthwise that I’m dealing with that makes [the lifestyle change] difficult.” “It was, okay, we’ve got acupuncture. We’ve got sleep. We’ve got the exercise, the MOVE…I suffer from chronic depression and anxiety [and] I just kind of felt overwhelmed.”
Access barriers	“Between my schedule, the distance it takes to get down here, it’s just impossible for me to come [to exercise class].” “I’m the caregiver for my husband… I just have to find time for myself, and sometimes that’s hard to do.”
Information	“I’m a little confused at this point on whether a vegetarian diet or a low carb diet is better…I think that’s probably my biggest confusion and I’m trying to get information related to that.” “I tried to go vegan, but I didn’t feel like I got the right nutrients… and I didn’t research it very well, so I just ate like frozen meals and a lot of veggies and fruit.”
COVID-19	“I wanted to try the gluten-free [diet]…I’ve been unable because the food lines in the grocery stores were crazy back then…So I said, okay, when this is over with, then I’ll do this.” “I joined the Y and that was very positive until the pandemic and all the gyms closed. So that hurt a lot.”
Personal attributes	“I’m just a master of procrastination.” “My sweet tooth is uncontrollable.” “Barriers, no not really. Just behavioral, personal behavioral patterns.”

Mental health–related barriers included anxiety, depression, PTSD, and general stress. These conditions impacted women Veterans’ motivation to sustain lifestyle changes, disrupted efforts to seek support or professional assistance, and, in some cases, fueled unhealthy behaviors, such as smoking, to relieve stress: “It’s not like I crave nicotine. I crave the 5 to 10 min of peace and quiet that I get because I can walk away from everyone…Because you can’t smoke anywhere except off by yourself. It’s not the nicotine; it’s the solitude… I’ve tried [to quit smoking] at least twice, three times in the past, and I’m still trying, but it’s that stress thing.” Some women felt overwhelmed by the experience of introducing a lifestyle change while already trying to address multiple co-occurring physical and mental health issues.

Women Veterans also noted access barriers that interfered with their ability to implement healthy lifestyle changes. Inadequate transportation and/or having to travel extensive distances to medical facilities or gyms limited women’s access to exercise or nutrition classes, weight loss programs, and professional dieticians/nutritionists. Busy work schedules and care-giving responsibilities limited the amount of time they could devote to exercise or planning healthier meals. One woman Veteran identified multiple challenges associated with implementing healthy eating habits, including not being able to find time to research new recipes and learn new cooking techniques: “It’s a complete learning experience…you’ve just got to cook a completely different way.” Women Veterans also mentioned insufficient information as a barrier to implementing dietary improvements, such as not knowing whether a vegetarian or low-carb diet was preferable and how to effectively transition to a vegan diet.

Of note, several women Veterans identified the COVID-19 pandemic as a barrier that frustrated their efforts to increase exercise (due to gym closures and class cancellations), change their diet (due to food shortages and long lines at the grocery store), and impaired their access to needed mental health resources. In contrast, a few women Veterans found that they had more time to reflect on and implement practices of self-care during the COVID-19 pandemic lockdown.

Finally, women Veterans attributed personal characteristics to their difficulties implementing changes, naming inconsistency, a tendency to procrastinate, “laziness,” negative thinking, and a lack of discipline as barriers to successfully implementing lifestyle changes. One woman Veteran, who described her collection of mental health practices as a “toolbox,” explained, “Sometimes I remember to open the toolbox. Sometimes I forget.” These women often attributed their ability to be successful to themselves alone, suggesting, “The only barrier was myself,” and “it really is my own fault from laziness.”

### Success Strategies

Women Veterans reported several strategies associated with success (Table 3). These strategies included the following: taking an incremental approach to accommodate their limitations and personal preferences; setting small, achievable goals; and implementing targeted provider recommendations. Women also reported achieving success through self-education (e.g., researching healthy eating techniques or symptom management), finding a compelling motivation, and engaging in mindfulness practices.

**Table 3:** Women Veterans’ Strategies to Successfully Implement Lifestyle Changes

Strategy	Exemplary quotes
Incremental approach	“I am a caregiver, but I’ve been trying to get out and at least walk, maybe like 15 min, 15 or 30 min, three times a week, outside of my regular everyday stuff…Instead of taking the elevator, try to take the stairs, stuff like that.” “I don’t eliminate any foods. Like I just had half a cookie that I’m sharing with my roommate. But I try to be more conscious…not have a bunch of it. Just have it in moderation.”
Achievable goals	“Don’t overwhelm myself…Don’t get on the treadmill like it’s a task. [Do] maybe 10, 15 min and allow yourself to stay on longer if you want to. If you don’t, that’s fine.” “[My goal is] just to maintain…I’m not going to put pressure on myself to lose weight at the moment, but just to try not to gain weight.”
Provider recommendations	“I was put on Chantix, and I was able to stop smoking that way… The smoking cessation people [at VA], they gave me little tips and tricks to help.” “[My provider] gave me a folder [with] some recommendations, especially about eating protein…It was really helpful. It kind of changed my lifestyle…she really took the time to educate me.”
Self-education	“I’ve taken the initiative to learn as much as I could about…what works and what doesn’t work for different medical conditions I have.” “It’s not like I picked one specific diet. I just kind of researched different diets and made it my own.”
Motivation	“I had a two-year-old granddaughter and a year-old grandson, and…he didn’t like to snuggle because I smelled [like smoke]…so I decided that's it, I’m done, and I have not smoked since.” “This is like kinda like a vain thing, but…I have like all these really nice clothes that I used to fit in and I don’t wanna buy bigger clothes.”
Mindfulness	“[The COVID-19 pandemic] allowed me to stop and to see…the impact of what I've been doing pre-COVID and how it has really been somewhat detrimental to my overall wellness…So it has been quite refreshing to be able to do the things that I always enjoyed doing in the past, and now allowing myself the time to say that I need to do it.” “I discovered the Fitbit a couple of years ago and it really…motivates me to move even more than I usually do…Actually, it’s helped me with my sleep too [and] with positive reinforcement.”

#### Incremental Approach

Women Veterans were able to address many of the barriers previously described, such as physical limitations, busy work schedules, and caregiving responsibilities, using an incremental approach in which they modified their lifestyle change efforts to create specific goals or modified steps. For example, when they did not have time for physical activity, women Veterans found ways to incorporate exercise into their daily routines, such as taking the stairs instead of the elevator or taking walking breaks at work. This incremental approach also involved accommodating personal preferences, such as adding healthier food choices without completely eliminating unhealthy foods they liked or scheduling a massage when they could not reduce stress through other means. Women Veterans also adopted a balanced attitude towards eating that was not rooted in restriction, elimination, or cycles of “yo-yo” dieting but instead involved making “conscious” decisions about what and how much to eat. One woman Veteran, who created her own meal plan based on existing nutritional guidance, food preferences, and her physical activity regimen, commented, “I took that mentality of let’s make it my own, let me figure out what works for me and then I’m more likely gonna stick with it.”

Underlying this strategy, women Veterans expressed considerable self-awareness with regard to what would and would not work for them. For example, women Veterans mentioned preferring tai chi to yoga or exercising with others instead of alone. These women frequently suggested knowing what it was that they had to do to be successful; it was simply a matter of putting that awareness into practice: “I’ve known what to do. It’s just a matter of doing it.”

#### Achievable Goals

Women Veterans also attributed their success to setting small, achievable goals. With regard to exercise, women Veterans described aiming for shorter intervals (10–15 min), shorter distances (1000–2000 steps), or a slower pace to meet their desired levels of physical activity. For weight loss, one woman Veteran mentioned maintaining, rather than trying to decrease, her weight. These strategies emerged from understanding their limits and accepting that their limits may fluctuate from day to day. One woman Veteran explained, “Maybe I have bad days, and during my bad days, you know, I still walk, but shorter… I don’t push it to the limit, because I know my limit…If I can’t [walk], okay. Maybe not today, but tomorrow…I think you just have to do what works best for you.”

#### Provider Recommendations

Targeted advice from providers that included specific suggestions on how to implement lifestyle changes, such as dietary substitutions, fitness apps, or smoking cessation tips, also helped women Veterans be successful in their efforts. One woman Veteran noted the importance of her provider’s own success in substituting zucchini noodles for more carbohydrate-heavy options, explaining, “I thought, wow, if even her children took to it that may be something I want to try.” Others highlighted the personalized nature of their providers’ advice, which was tailored to their unique barriers and lifestyle preferences. For example, a Veteran struggling with gastro-intestinal issues, noted, “She was able to tell me, ‘Okay, you can go on this website and look for anti-inflammatory foods, and that will help.’ And it’s just little things like that, like, you know?” Alongside targeted suggestions, women Veterans also appreciated educational materials from their providers, such as brochures with detailed information about protein intake and portion sizes.

#### Additional Strategies: Self-Education, Motivation, Mindfulness

Women Veterans reported additional strategies to help them successfully implement lifestyle changes; they included self-education, finding a compelling motivation, and mindfulness. To better inform their approach, women Veterans described conducting research on their own to determine healthy eating techniques, exercise regimens, or symptom management strategies that aligned with their needs and preferences and would not conflict with physical health limitations or other barriers. These women frequently mentioned having a prior interest in, or having previously conducted research on, health-promoting behaviors, and drew on this prior knowledge to inform current lifestyle change efforts. In addition, these women Veterans often described themselves as “self-starters” who were accustomed to taking proactive measures to improve their health. Women Veterans also mentioned the importance of having a compelling motivation to help them succeed in sustaining a lifestyle change. Motivations included improving their health for the benefit of those who relied on them (e.g., children, significant others, or pets); having a family history of CVD; wanting to alleviate existing health symptoms and/or prevent illness and frailty; and not wanting to have to buy new clothes due to weight gain. Finally, women Veterans mentioned mindfulness practices, including taking time for self-reflection or tracking their efforts (e.g., number of steps recorded on a fitness app, numbers of hours of sleep tracked, or improved blood pressure readings), as facilitators that contributed to their success. These practices provided positive reinforcement, encouraging women Veterans to sustain their lifestyle change efforts.

## DISCUSSION

Women Veterans engaged in a wide range of lifestyle changes to improve their cardiovascular health. While most changes focused on exercise and diet, women also aimed to improve their sleep, mental health, and medication adherence, and tried to quit smoking. Despite numerous reported barriers, the women Veterans in our study expressed high levels of self-awareness regarding strategies that would help them successfully implement the lifestyle changes they wished to make. The most commonly mentioned strategies were adopting an incremental approach, setting achievable health goals, and implementing targeted suggestions from providers.

Women Veterans who adopted an incremental approach often modified their efforts to align with personal lifestyle needs and preferences. This process allowed women to partition a larger lifestyle goal, such as losing weight, into manageable steps that were specific, measurable, action-oriented, reliable, and often time-limited—in line with VA-supported “SMART” goals.^23^ This personalized, proactive approach is also embraced by a “whole health” philosophy underlying lifestyle medicine, which promotes the development of patient-driven health goals to prevent and treat chronic disease.^24–26^ Centering patients’ goals and priorities within lifestyle change efforts can result in improved patient engagement and health outcomes.^27^ This approach is particularly valuable for patients with multiple morbidities and/or psychosocial complexities, who may struggle to adhere to inflexible behavior change strategies.^28^ Additionally, Veterans have higher rates of mental health disorders, substance use disorders, and chronic health conditions compared to civilian populations^29^ and have identified physical and chronic health conditions as barriers to increasing physical activity^30,31^ and improving diet.^32^ Patient-centered modifications may therefore prove particularly valuable for Veteran populations seeking to adopt and adhere to lifestyle changes to reduce CVD risk.

Setting small, achievable goals also helped women Veterans in our study successfully implement lifestyle changes. Prior research has shown that a “small change” approach to weight loss—emphasizing incremental, gradual improvements—can help Veterans achieve and sustain desired weight loss outcomes over time.^33,34^ Our findings suggest that such an approach may apply to a wider range of lifestyle change efforts to reduce cardiovascular risk and may be particularly beneficial for women Veterans, who face unique barriers to lifestyle intervention adherence.^11^ Additionally, our findings encourage providers to develop patient-centered goals rather than adopting a prescriptive, one-size-fits-all approach to lifestyle change implementation.

Our study also highlighted the important role providers play in supporting patients’ lifestyle change efforts, namely by offering additional education and specific guidance around implementation techniques. Primary care providers may be a critical resource for Veterans with limited access to, or low motivation to participate in, lifestyle intervention programs and services, such as nutrition classes or weight loss programs.^35^ However, primary care providers may have limited time, a lack of awareness of up-to-date CVD prevention guidelines, limited proficiency in counseling patients to support lifestyle change implementation, and lack of vetted educational information and available resources.^16,36,37^ Our findings suggest the need to strengthen support for providers, who directly impact patient success through targeted suggestions and education that helps patients put recommendations into action.

Other strategies that facilitated women Veterans’ lifestyle change efforts included self-education, having a compelling motivation, and mindfulness. Women Veterans who addressed knowledge gaps in how to implement specific lifestyle changes frequently described themselves as pro-active and expressed a willingness to take initiative to improve their cardiovascular health. While developing self-efficacy may promote long-term lifestyle change success,^38,39^ further research is needed on how to better support Veterans who struggle to take a proactive role in their cardiovascular care. Structural factors, such as low income and food insecurity, may override any other healthcare priority setting due to basic needs not being met and may also impede access to knowledge about how to prepare nourishing food, creating additional barriers for lifestyle change implementation.^40,41^ Low motivation is another well-known barrier to lifestyle intervention adherence, particularly among patients seeking to lose weight and increase physical activity.^42–44^ Using motivational interviewing techniques, providers may help patients clarify their behavior change goals and build self-confidence, leading to improved engagement in and sustainment of lifestyle change interventions.^45,46^ Additionally, the ability to monitor lifestyle change efforts through mobile weight loss applications, fitness trackers, or journaling has shown favorable results among obese and overweight Veterans^30^ as well as Veterans with prediabetes.^47^ Our findings support that connecting women Veterans with resources to track their efforts may provide positive reinforcement and contribute to long-term success. Finally, our findings identified mixed impacts of the historic events such as the COVID-19 pandemic on healthy lifestyle behaviors, with some women Veterans identifying increased barriers to lifestyle change implementation and others noting increased opportunities to devote to exercise or meal-planning. Similar findings have been noted elsewhere, with individuals adopting or improving healthy lifestyle habits, such as quitting smoking and increasing physical activity, during the COVID-19 pandemic.^48^

### Limitations

First, our sample was drawn from five VA primary care facilities; as a result, our findings are not generalizable to all Veterans or VA settings. Second, our study is limited by self-selection bias: the women Veterans who volunteered to participate in interviews may have significantly different barriers and facilitators compared to those who declined. For example, the women Veterans in our sample expressed considerable self-awareness as well as prior experience making lifestyle changes. Success strategies may differ for women Veterans attempting to make lifestyle changes for the first time.

## CONCLUSION

Identifying patient-reported strategies associated with successful efforts to implement lifestyle changes can inform the tailoring of effective interventions to reduce CVD risk among women Veterans. Our study highlights the importance of harnessing women Veterans’ self-awareness to guide personalized, realistic strategies that maximize success. For women Veterans who “know what to do” but need additional implementation support, identifying incremental approaches, achievable goals, and targeted recommendations may help bridge the gap between knowledge and action, or action and sustainability.

Providers play a critical role in supporting women Veterans’ efforts. Encouraging women Veterans to reflect on factors that may facilitate or impede their lifestyle change efforts (e.g., barriers, personal preferences) and adopting evidence-based practices, such as motivational interviewing, when counseling women on lifestyle change implementation, may improve patients’ self-efficacy and long-term adherence to lifestyle changes. Ongoing patient-provider collaboration, including clear communication, identifying small steps, and honoring patients’ goals using evidence-based tools or recommendations, is essential for generating behavior change strategies that promote enduring success.

## Data Availability

Final data sets underlying this publication cannot be shared outside of the VA due to VA data policies designed to protect patient privacy.
